# Modified Flory–Rehner Theory Describes Thermotropic Swelling Transition of Smart Copolymer Microgels

**DOI:** 10.3390/polym14101999

**Published:** 2022-05-13

**Authors:** Simon Friesen, Sergej Kakorin, Thomas Hellweg

**Affiliations:** Department of Chemistry, Physical and Biophysical Chemistry, Bielefeld University, Universitätsstr. 25, 33615 Bielefeld, Germany; simon.friesen@uni-bielefeld.de (S.F.); sergej.kakorin@uni-bielefeld.de (S.K.)

**Keywords:** thermoresponsive copolymer microgels, NIPAM, NNPAM, NIPMAM, Flory–Rehner theory, swelling behavior, cooperativity

## Abstract

In the present article, we use an improved Flory–Rehner theory to describe the swelling behavior of copolymer microgels, where the interaction parameter is modeled by a Hill-like equation for a cooperative thermotropic transition. This description leads to very good fits of the swelling curves of the copolymer microgels at different comonomer contents (30 mol%, 50 mol% and 70 mol%) obtained by photon correlation spectroscopy. Fixed parameters, which are universally applicable for the respective monomers given in our previous work, are used to fit the swelling curves. The analysis of the swelling curves yields physically reasonable and meaningful results for the remaining adjustable parameters. The comonomer content of the statistical copolymer microgels poly(NNPAM-co-NIPAM), poly(NIPAM-co-NIPMAM) and poly(NIPMAM-co-NNPAM) is determined by nuclear magnetic resonance spectroscopy and is in agreement with the nominal comonomer feed used in the synthesis. To investigate the volume phase transition at a molecular level, swelling curves are also measured by Fourier transformation infrared spectroscopy. The obtained swelling curves are also fitted using the Hill-like model. The fits provide physically reasonable parameters too, consistent with the results from photon correlation spectroscopy.

## 1. Introduction

Thermoresponsive microgels are colloidal particles, comprising gel networks internally, that abruptly change their volume at the so-called volume phase transition temperature (VPTT) which coresponds to the lower critical solution temperature (LCST) of the used polymer [1,2]. Due to this feature, thermoresponsive microgels are promising for example as drug delivery systems [3,4,5,6], as carriers for enzymes [7,8,9] or catalytic nanoparticles [10,11,12,13], or as responsive surface coating [14,15]. The large number of potential applications of thermoresponsive microgels have led to intensive research on these so-called “smart” systems in recent decades [1,2,16,17,18,19,20,21,22,23,24]. Depending on application, it is advantageous to tune the VPTT of microgels. Therefore, the choice of monomers for synthesis of microgels is crucial. The most intensively studied systems are *N*-isopropylacrylamide based microgels which show a VPTT of about 32 °C [1]. Alternative monomers are *N-n*-propylacrylamide (VPTT ≈ 22 °C) [25] and *N*-isopropylmethacrylamide (VPTT ≈ 45 °C) [26]. By copolymerizing random thermoresponsive comonomers, the volume phase transition (VPT) can be shifted to higher or lower temperatures, for example, by varying the comonomer content [27]. A variety of thermoresponsive copolymer microgels have been synthesized in which the VPTT could be varied over a wide range [18,27,28,29,30]. For example, in previous works [18,27,28,30] based on *N-n*-propylacrylamide (NNPAM), *N*-isopropylacrylamide (NIPAM) and *N*-isopropylmethacrylamide (NIPMAM), thermoresponsive microgels with variable VPTT between 22 °C and 45 °C were synthesized in a controlled and systematic manner by varying the comonomer content. It was shown that the VPTT of copolymer microgels depends on the composition and choice of monomers.

In the article at hand, we use a thermodynamic approach to explain the swelling behavior of thermoresponsive copolymer microgels. The swelling behavior of smart microgels is usually described using the classical Flory–Rehner theory [31]. Since the interaction parameter χ of the original Flory–Rehner theory is not always adequate to trace the experimental swelling curves, series expansions of χ with respect to the polymer volume fraction ϕ have been proposed [32,33]. However, the coefficients of such series expansions are physically difficult to interpret. Furthermore, the series expansions do not take into consideration the cooperativity of the volume phase transition coming from the chemical coupling of polymer chains [34,35,36,37]. Tiktopul et al. [34] investigated the cooperativity of the coil-globule transition of poly(NIPAM) by microcalorimetry and have shown that the polymer does not undergo a globule-coil transition according to the all-or-none mechanism. The process is rather gradually cooperative. Leite et al. [38] and Friesen et al. [35,36] proposed a Hill-like model that accounts for cooperativity of the volume phase transition. However, it is an even greater challenge to describe the swelling behavior of copolymer microgels thermodynamically and at least for higher comonomer contents standard models for χ fail. This is especially true when the polymerized comonomer does not exhibit a LCST [29,39]. Despite of the large number of syntheses of statistical copolymer microgels, theoretical descriptions of the VPT of these systems are sparse [29]. A general extension of the Flory–Rehner theory was proposed by Godbole et al. [40] which can be applied to describe the swelling behavior of copolymer microgels. In this paper the swelling properties of synthesized homopolymer microgels based on poly(NNPAM) (PNNPAM), poly(NIPAM) (PNIPAM), and poly(NIPMAM) (PNIPMAM) and nominally statistic copolymer microgels based on poly(NNPAM-co-NIPMAM), poly(NNPAM-co-NIPAM) and poly(NIPAM-co-NIPMAM) are investigated at a mesoscale level by photon correlation spectroscopy (PCS) and at a molecular level by Fourier transformation infrared spectroscopy (FTIR) in a temperature dependend manner. To quantify the amount of the different monomers present in the copolymer microgels ^1^H-NMR spectra were recorded. We use a general extension of the Flory–Rehner theory proposed by Godbole et al. [40] combined with our recently introduced Hill-like model for the interaction parameter χ to quantify the $R_{H}\left(t\right)$-swelling curves of copolymer microgels obtained by PCS (please note that we use *t* as symbol for the temperature in Celsius to distinguish from absolute temperatures in Kelvin). The thermodynamic analysis uses such parameters as the volume phase transition temperature $VPTT_{fit}$, the polymer volume fraction in the reference state $\phi_{0}$ and the number of segments (monomers) between two crosslinkers $N_{Seg}$ at different mole fractions of comonomers *x* in the microgels. Swelling curves obtained by FTIR were fitted using the Hill-like model.

## 2. Theory

### 2.1. Flory–Rehner Theory for Copolymer Microgels

For the description of the swelling behavior of copolymer microgels, the classical Flory–Rehner theory for homopolymer microgels must be extended, since the different interactions of the individual comonomers must be taken into account. Such an extension of the Flory–Rehner theory was proposed by Godbole et al. [40]. For a copolymer microgel with two comonomers *a* and *b*, the elastic contribution to the osmotic pressure $\Pi_{el}$ is given by:

(1) $$\Pi_{el}=\frac{N_{c}k_{B}T}{V_{0}}\left[\frac{\phi_{a}+\phi_{b}}{2\phi_{0}}-{\left(\frac{\phi_{a}+\phi_{b}}{\phi_{0}}\right)}^{1/3}\right]$$

where $k_{B}$ is the Boltzmann constant, *T* is the temperature and $\phi_{a}$ and $\phi_{b}$ are the volume fraction of comonomer *a* and *b*, respectively. $V_{0}$ is the volume of the microgel in the collapsed state and $N_{c}$ is the number of polymer chains. The mixing contribution to the osmotic pressure $\Pi_{mix}$ for a copolymer microgel with two comonomers *a* and *b* is given by [40,41]:

(2) $$\Pi_{mix}=-\frac{N_{A}k_{B}T}{\nu_{S}}\left[\ln\phi_{s}+\left(1-\phi_{s}\right)+\left(1-\phi_{s}\right)\left(\phi_{a}\chi_{s,a}+\phi_{b}\chi_{s,b}\right)\right]$$

where $\phi_{s}$ is the volume fraction of the solvent *s*, $\chi_{s,a}$ and $\chi_{s,b}$ are the respective interaction parameters for characterizing the interaction between solvent and comonomer *a* and *b*, respectively and the interaction parameter $\chi_{a,b}$ which describes the interactions between comonomers *a* and *b*. As in the standard Flory–Rehner theory, the contributions to the free energy change ΔF are assumed to be a sum of the mixing contribution $\Delta F_{mix}$ and the elastic contribution $\Delta F_{el}$. If $\Pi_{el}$ (Equation (1)) and $\Pi_{mix}$ (Equation (2)) are equal in swelling equilibrium, Equation (3) is obtained for the thermodynamic description of the swelling behavior of a binary copolymer microgel [40].

(3) $$\begin{array}{c} \frac{N_{A}k_{B}T}{\nu_{S}}\left[\ln\phi_{s}+\left(1-\phi_{s}\right)+\left(1-\phi_{s}\right)\left(\phi_{a}\chi_{s,a}+\phi_{b}\chi_{s,b}\right)\right]\\ +\frac{N_{c}k_{B}T}{V_{0}}\left[{\left(\frac{\phi_{a}+\phi_{b}}{\phi_{0}}\right)}^{1/3}-\frac{\phi_{a}+\phi_{b}}{2\phi_{0}}\right]=0\;. \end{array}$$

with the relationships for the number of segments between two crosslinkers $N_{Seg}=\left(V_{0}N_{A}\phi_{0}\right)/\left(\nu_{s}N_{c}\right)$ (where $N_{A}$ is the Avogadro constant and $\nu_{s}$ is the molar volume of the solvent) and the total polymer volume fraction $\phi =\phi_{a}+\phi_{b}=1-\phi_{s}$, the Flory–Rehner Eq. for copolymer microgels is given by:

(4) $$\ln\left(1-\phi \right)+\phi +\phi \left(1+\phi_{a}\chi_{s,a}+\phi_{b}\chi_{s,b}\right)+\frac{\phi_{0}}{N_{Seg}}\left[{\left(\frac{\phi }{\phi_{0}}\right)}^{1/3}-\frac{\phi }{2\phi_{0}}\right]=0\;.$$

If the mole fractions $x_{a}$ and $x_{b}$ of the comonomers are known then the volume fractions of $\phi_{a}$ and $\phi_{b}$ are given by:

(5) $$\begin{array}{c} \phi_{a}=x_{a}\phi \;\phi_{b}=x_{b}\phi \;. \end{array}$$

Using the Equation (5) and replacing the polymer volume fraction ϕ by the hydrodynamic radius $R_{H}=R_{H,0}{\left(\phi_{0}/\phi \right)}^{1/3}$ in Equation (4), the modified Flory–Rehner Eq. we use in this paper to describe copolymer microgels is given by:

(6) $$\begin{array}{c} \ln\left(1-{\left(\frac{R_{H,0}}{R_{H}\left(t\right)}\right)}^{3}\phi_{0}\right)+{\left(\frac{R_{H,0}}{R_{H}\left(t\right)}\right)}^{3}\phi_{0}+{\left(\frac{R_{H,0}}{R_{H}\left(t\right)}\right)}^{6}\phi_{0}^{2}\left(x_{a}\chi_{s,a}+x_{b}\chi_{s,b}\right)\\ +\frac{\phi_{0}}{N_{Seg}}\left[\frac{R_{H,0}}{R_{H}\left(t\right)}-\frac{1}{2}{\left(\frac{R_{H,0}}{R_{H}\left(t\right)}\right)}^{3}\right]=0 \end{array}$$

where $R_{H,0}$ is the hydrodynamic radius in the collapsed state and $R_{H}\left(t\right)$ is the hydrodynamic radius at temperature *t*. The homopoylmer systems poly(NNPAM), poly(NIPAM) and poly(NIPMAM) are described by the Flory–Rehner theory for homopolymers:

(7) $$\begin{array}{c} \ln\left(1-{\left(\frac{R_{H,0}}{R_{H}\left(t\right)}\right)}^{3}\phi_{0}\right)+{\left(\frac{R_{H,0}}{R_{H}\left(t\right)}\right)}^{3}\phi_{0}+{\left(\frac{R_{H,0}}{R_{H}\left(t\right)}\right)}^{6}\phi_{0}^{2}\chi \\ +\frac{\phi_{0}}{N_{Seg}}\left[\frac{R_{H,0}}{R_{H}\left(t\right)}-\frac{1}{2}{\left(\frac{R_{H,0}}{R_{H}\left(t\right)}\right)}^{3}\right]=0 \end{array}$$

### 2.2. Hill-like Model for the Interaction Parameter

The Hill-like model describes the interaction parameter χ by taking into account the cooperativity of the binding of solvent molecules to the polymer. The state before and after the thermotropic transition is described by the reaction:

(8) $$\left.PS_{\nu }⇄P+\nu S\right.$$

where ν is the stoichiometric coefficient of the reaction (Equation (8)) and represents the number of solvent molecules (here water) which leave the microgel per polymer segment. The symbol $PS_{\nu }$ denotes the aggregate state below the VPTT. *P* and νS denote the states of the polymer and solvent above the VPTT, respectively.

The Hill model can be considered as a useful empirical approximation of cooperative ligand binding on a receptor, especially in the cases of large positive cooperativity [42]. Note, that in our case the Hill coefficient is varying between 10–20, suggesting extremely positive cooperativity of the temperature induced volume phase transition in polymer micro-gel-particles. The Hill model requires little a priori knowledge about the details of physical-chemical mechanism of water binding on polymer segments. A deeper insight into underlying polymer-water interaction can be won, e.g., by molecular dynamic computer simulations [43,44]. Using the Hill-like model, Leite et al. [38] and Friesen et al. [35] were able to describe the swelling behavior of various homopolymer microgels. We have shown in a previous work that the Hill-like model for the interaction parameter, yields physically meaningful parameters in contrast to the original approach of Flory and to series expansions of χ [36]. The Hill-like model is given by:

(9) $$\chi \left(t\right)=\chi_{0}+a\left(t-t_{a}\right)+b\frac{t_{rel}^{\nu }}{t_{rel}^{\nu }+K}$$

where $\chi_{0}$ is the value of the χ parameter at $t=t_{a}$, $t_{a}$ is the first temperature data point, and $t_{e}$ is the last (end) temperature point of the data set, *a* is the slope of the baseline, *b* is the dimensionless amplitude parameter of the Hill transition, *K* is the half-saturation constant, $t_{0.5}$ is the half-temperature, ν is the Hill parameter and $t_{rel}\left(t\right)=\left(t-t_{a}\right)/\left(t_{e}-t_{a}\right)$ is the relative temperature. Note that in the thermotropic transition the concentration of the species is replaced by a relative temperature $t_{rel}$ that changes in the range $0\leq t_{rel}\left(t\right)\leq 1$. Since the half-temperature $t_{0.5}$ corresponds to the VPTT, *K* can be described by [35]:

(10) $$K={\left(\frac{VPTT_{fit}-t_{a}}{t_{e}-t_{a}}\right)}^{\nu }\;.$$

The interaction parameters $\chi_{s,a}$ and $\chi_{s,b}$ are given by the Hill-like model by:

(11) $$\chi_{s,i}\left(t\right)=\chi_{i,0}+a_{i}\left(t-t_{a}\right)+b_{i}\frac{t_{rel}^{\nu_{i}}}{t_{rel}^{\nu_{i}}+{\left(\frac{VPTT_{fit}-t_{a}}{t_{e}-t_{a}}\right)}^{\nu_{i}}}$$

where the index *i* stands for a polymer component which in this work can be NNPAM, NIPAM or NIPMAM. Hence, for the present case the total χ parameter is the sum of the mole fraction $x_{i}$ weighted $\chi_{s,i}$ values.

## 3. Results and Discussion

### 3.1. ^1^H-NMR

**Figure 1:**
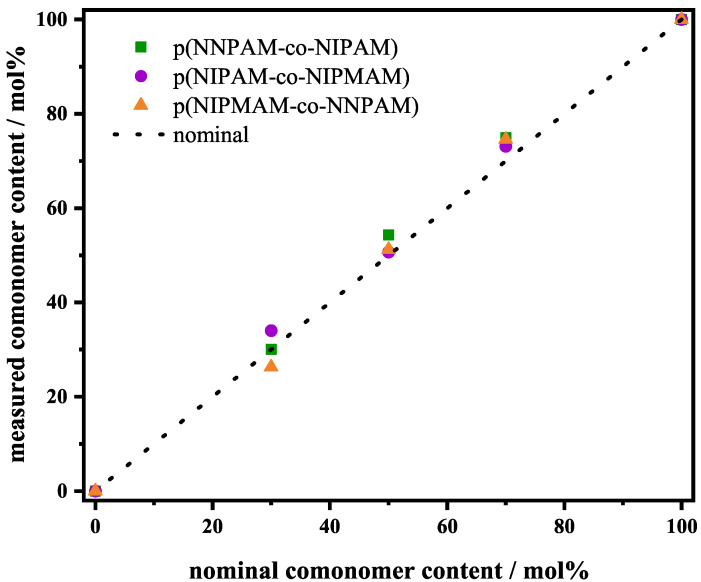
Measured comonomer content vs. nominal comonomer content for poly(NNPAM-co-NIPAM) (squares), poly(NIPAM-co-NIPMAM) (circles) and poly(NIPMAM-co-NNPAM) (triangles) microgels. The copolymerization ratio was calculated from the ^1^H-NMR spectra. The dashed line represents the correlation between nominal monomer feed and measured real incorporated comonomer content.

The real incorporated comonomer content was quantified by means of ^1^H-NMR spectra (Figure 1). A three-fold measurement was performed for this quantification, see  Table S1. The detailed analysis of the spectra is summarized in Supplementary Materials (SM).

For all fits, the molar fraction $x_{i}$ determined by ^1^H-NMR (Table 1) of the respective components was used instead of the value of the nominal monomer feed based molar fraction.

### 3.2. Analysis of the $R_{H}\left(t\right)$-Swelling Curves

To describe the volume phase transition of the copolymer systems poly(NNPAM-co-NIPAM), poly(NIPAM-co-NIPMAM) and poly(NIPMAM-co-NNPAM) the Flory-Rehner Equation (6) for copolymers and the Hill-like Equation (11) for the interaction parameter $\chi_{s,i}$ were used. The thermodynamic description of the swelling behavior of the homopolymer systems poly(NNPAM), poly(NIPAM) and poly(NIPMAM) is given by the Flory–Rehner Equation (7) for homopolymers with the Hill-like Equation (9) for the calculation of the interaction parameter χ.

**Figure 2:**
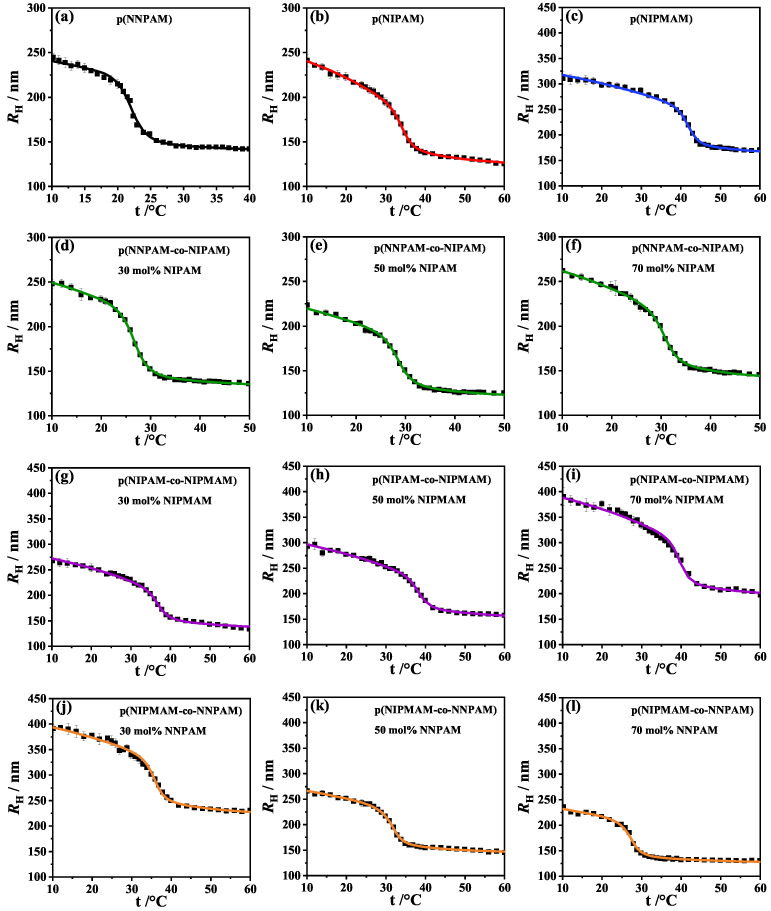
Hydrodynamic radius $R_{H}$ vs. temperature *t* of the homopolymer systems poly(NNPAM) (**a**), poly(NIPAM) (**b**) and poly(NIPMAM) (**c**) and of the copolymer systems poly(NNPAM-co-NIPAM) (**d**–**f**), poly(NIPAM-co-NIPMAM) (**g**–**i**) and poly(NIPMAM-co-NNPAM) (**j**–**l**) at different comonomer contents (30 mol%, 50 mol% and 70 mol%). Squares are experimental data, solid lines represent the fitting curves. $R_{H}\left(t\right)$ of the homopolymer systems was calculated using the Flory–Rehner Equation (7) for homopolymers. For the fit of the hydrodynamic radii $R_{H}\left(t\right)$ of the copolymer systems the modified Flory–Rehner Equation (6) for copolymer networks was used. The interaction parameter $\chi_{s,i}$ was calculated with the Hill-like Equation (11) for both monomers, respectively. A nearly perfect fit to the experimental data was achieved for all microgel systems.

The theoretically calculated hydrodynamic radii $R_{H,fit}\left(t,\phi_{0},N_{Seg},VPTT_{fit}\right)$ as a function of temperature *t* were fitted to the experimentally determined swelling curves $R_{H,exp}\left(t\right)$ obtained by PCS, see Figure 2. The hydrodynamic radius of the particle $R_{H}\left(t\right)$ is a free variable, which can be found by solving the nonlinear Equation (6) for copolymer microgels and the nonlinear Equation (7) for homopolymer microgels. For the fitting procedure the software Mathcad Prime 6.0 was used which solves the nonlinear equations by using the Levenberg-Marquardt algorithm. For solving the Equations (6) and (7), respectively, the fitting parameters $\phi_{0}$, $N_{Seg}$ and $VPTT_{fit}$ were varied. The parameters for the homopolymer systems $\chi_{0}$, *a*, *b* and ν were used from the previous work [35], see Table 2.

The parameters $\chi_{0}$, *a* and *b* are unique for the given homopolymer type and do not depend on the cross-linker *N,N’*-methylenebisacrylamide (BIS) or initiator ammonium persulfate (APS) concentrations used [35]. On the other hand, the number of water molecules ν leaving the gel at the volume phase transition per polymer segment, depends on the BIS concentration. Here, ν at 10 mol% was taken from the previous work [35] for the respective components. For the copolymer systems, the parameters $\chi_{0,i}$, $a_{i}$, $b_{i}$ and $\nu_{i}$ were also taken from the previous work [35] for the respective comonomers, see Table 2. For the copolymer systems as well as for the homopolymer systems the fitting parameters $\phi_{0}$, $N_{Seg}$ and $VPTT_{fit}$ were obtained, see Table 1. To evaluate the quality of the fits, ${\left(chi\right)}^{2}$ values are calculated by [45]:

(12) $${\left(chi\right)}^{2}=\sum_{t_{a}}^{t_{e}}\frac{{\left(R_{H,exp}\left(t\right)-R_{H,fit}\left(t,\phi_{0},N_{Seg},VPTT_{fit}\right)\right)}^{2}}{R_{H,fit}\left(t,\phi_{0},N_{Seg},VPTT_{fit}\right)}$$

where $R_{H,exp}\left(t\right)$ is the experimentally determined curve and $R_{H,fit}\left(t,\phi_{0},N_{Seg},VPTT_{fit}\right)$ is the fitted curve. For all fits, very small ${\left(chi\right)}^{2}$-values (Equation (12)) ranging from 0.3 nm to 2.7 nm were obtained for swelling curves with 34 data points, see Table 1.

Figure 2 shows that the fits are almost perfect in all cases which is also indicated by the small ${\left(chi\right)}^{2}$ values. All $R_{H}\left(t\right)$-swelling curves show only one continuous volume phase transition which is an indication that the volume phase transition is cooperative and the comonomer is statistically distributed in the microgel. Since all chains are chemically coupled, a collapse of one chain induces the volume phase transition in the whole polymer network. FTIR results also show only one volume phase transition (see sub-Section 3.3) and support this assumption that the volume phase transition is cooperative. The statistical distribution is also supported by the comonomer contents determined with ^1^H-NMR, see Figure 1 and Table 1. The successful fitting of the swelling curves with the fixed parameters $\chi_{0,i}$, $a_{i}$, $b_{i}$ and $\nu_{i}$ from the previous work [35] shows once more that these parameters are universally applicable for the respective monomers used. Hence, we believe that they can also be applied for the thermodynamic description of the swelling behavior of other microgels. It is remarkable that the swelling behavior of copolymer microgels can be fitted with just three fitting parameters. Furthermore, the fits also provide physically meaningful results.

**Figure 3:**
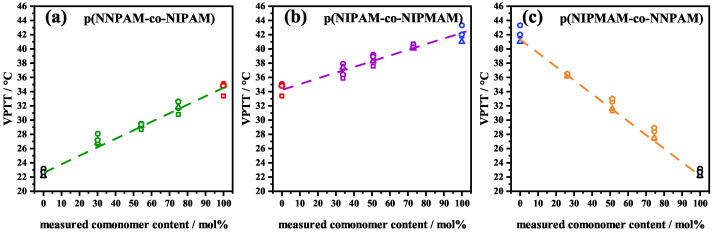
Volume phase transition VPTT vs. measured comonomer content of homopolymer systems poly(NNPAM) (black), poly(NIPAM) (red) and poly(NIPMAM) (blue) and copolymer systems poly(NNPAM-co-NIPAM) (green) (**a**), poly(NIPAM-co-NIPMAM) (purple) (**b**) and poly(NIPMAM-co-NNPAM) (orange) (**c**). $VPTT_{fit}$ (circle) was obtained from the fit of the $R_{H}\left(t\right)$-swelling curves; $VPTT_{IP}$ (square) was obtained from the fit of the $R_{H}\left(t\right)$-swelling curves $VPTT_{fit}$ (pentagon) was obtained from the fit of the ${\tilde{\nu }}_{\max}\left(t\right)$- swelling curves and $VPTT_{IP}$ (triangle) was obtained from the inflection point of the ${\tilde{\nu }}_{\max}\left(t\right)$-swelling curves. The VPTT follows a linear trend between the VPTT of the homopolymer systems. The dashed lines are guides to the eye.

The parameter $VPTT_{fit}$ determined by the fits gives physically reasonable values which are just 1 to 2 °C higher than $VPTT_{IP}$ determined by the inflection point of the experimental swelling curve (Table 1 and Figure 3).This is expected since the inflection point method slightly underestimates the VPTT. For the polymer volume fraction in the reference state $\phi_{0}$, values between 0.71 and 0.89 were found which are consistent with other studies [38,46,47,48,49]. The average degree of polymerization $N_{Seg}$ depends on the crosslinker concentration BIS. Since the same concentration of crosslinker was used in all microgel syntheses, the values for $N_{Seg}$ should not differ significantly from each other.

**Figure 4:**
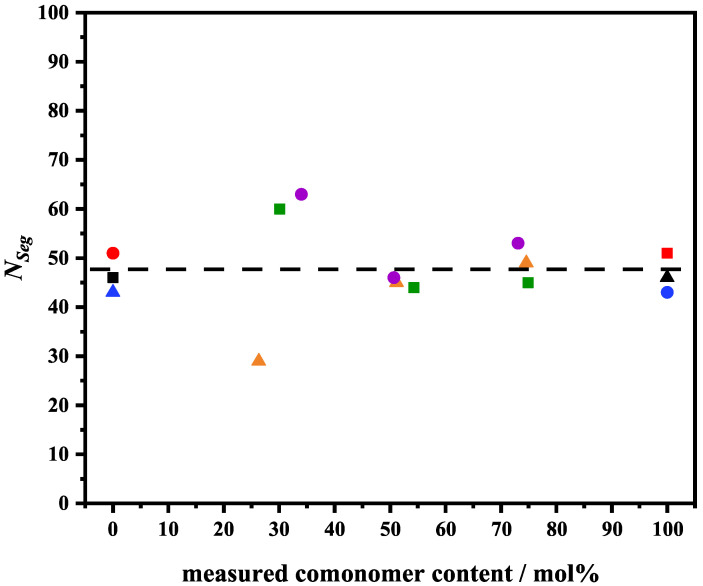
Average degree of polymerization $N_{Seg}$ vs. measured comonomer content poly(NNPAM-co-NIPAM) (squares), poly(NIPAM-co-NIPMAM) (circles) and poly(NIPMAM-co-NNPAM) (triangles). The dashed line marks the mean at 48.

As expected, the values for $N_{Seg}$ are very close and have an average value of 48 (Table 1 and Figure 4) which is also consistent with previous studies [35,36,38,49,50]. The differences between the $N_{Seg}$ values might be due to the different distributions of the cross-linker in the particular microgel particles.

The $\nu_{i}$ indicates the number of water molecules leaving the gel per polymer segment at the volume phase transition. Since the polymer chains in the copolymer microgel do not consist of only one type of monomer, the interaction parameter $\chi_{s,i}$ is weighted by the monomer content $x_{i}$ used. The overall description of the interaction between polymer and solvent (water in our case) is composed of the sum of the interaction parameters of the respective monomers weighted by the respective proportions. The total number of water molecules leaving the gel per segment at the volume phase transition $\nu_{total}$ is the sum of the Hill parameters $\nu_{i}$ weighted by the mole fraction of the respective monomers (Equation (13)).

(13) $$\nu_{total}=\sum_{i}x_{i}\nu_{i}$$

**Figure 5:**
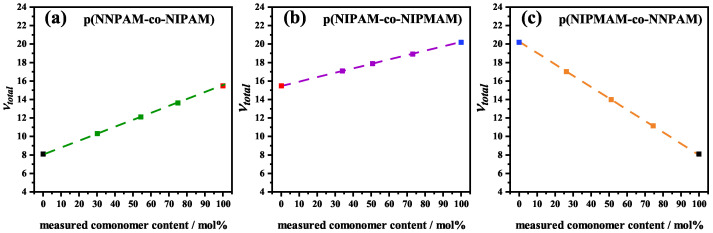
Total number of water molecules leaving the gel per segment at the volume phase transition $\nu_{total}$ vs. measured comonomer content of homopolymer systems poly(NNPAM) (black), poly(NIPAM) (red) and poly(NIPMAM) (blue) and copolymer systems poly(NNPAM-co-NIPAM) (green) (**a**), poly(NIPAM-co-NIPMAM) (purple) (**b**) and poly(NIPMAM-co-NNPAM) (orange) (**c**). $\nu_{total}$ was calculated with Equation (13). $\nu_{total}$ follows a linear trend between the ν values of the homopolymer systems. The dashed lines are guides to the eye.

The linear relationship between $\nu_{total}$ and the comonomer content (Figure 5) corresponds to the fact that the hydrophilicity of the copolymer microgels changes linearly with the comonomer content, which is also reflected in the linear change of the VPTT with $x_{i}$, see Figure 3. The monomer NIPAM is the most hydrophilic and therefore binds the most water molecules of the monomers used here. NNPAM is more hydrophobic than NIPAM and binds the fewest water molecules. Accordingly, NIPMAM releases more water molecules than NIPAM and NNPAM at the volume phase transition. With the increase of the more hydrophilic monomer, the number of water molecules leaving the gel per segment at the volume phase transition also increases linearly, see Figure 5.

### 3.3. Analysis of the FTIR-Swelling Curves

**Figure 6:**
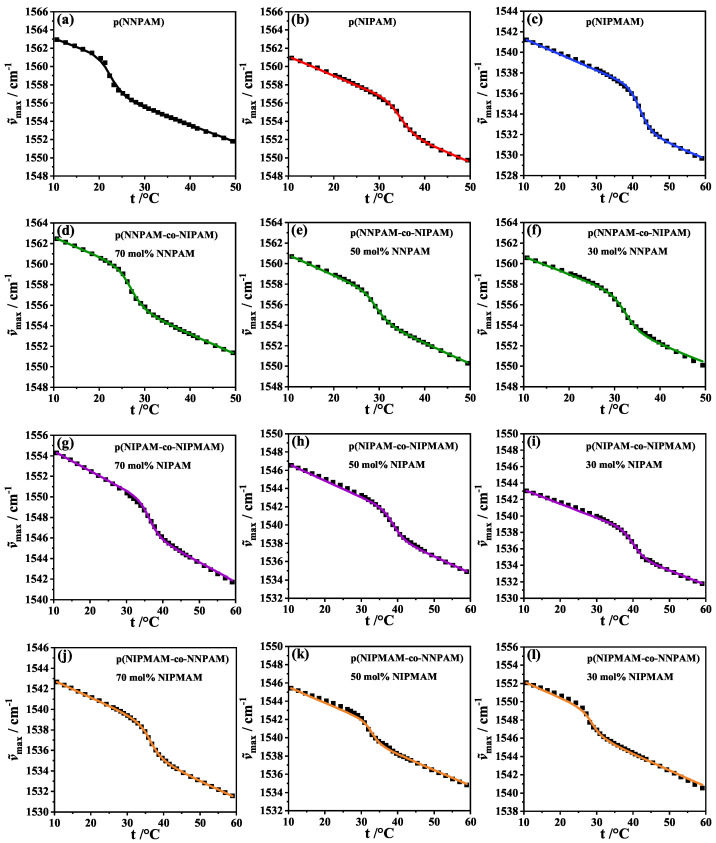
Maximum of the δ(N−H)-bending vibration band ${\tilde{\nu }}_{\max}$ vs. temperature *t* of homopolymer systems poly(NNPAM) (**a**), poly(NIPAM) (**b**) and poly(NIPMAM) (**c**) and of copolymer systems poly(NNPAM-co-NIPAM) (**d**–**f**), poly(NIPAM-co-NIPMAM) (**g**–**i**) and poly(NIPMAM-co-NNPAM) (**j**–**l**) at different comonomer contents (30 mol%, 50 mol% and 70 mol%). Squares are experimental data, solid lines represent the fitting curves. ${\tilde{\nu }}_{\max}$ of the homopolymer and copolymer systems are fitted using the Hill-like Equation (14). A nearly perfect fit to the experimental data was achieved for all microgel systems.

Insight into the local interaction behavior between solvent and polymer segments of the network is obtained via scrutinizing the N−H bond. The maximum of the δ(N−H)-bending vibration band, ${\tilde{\nu }}_{\max}\left(t\right)$ as a function of temperature *t* is plotted in Figure 6. A weakening of the hydrogen bond leads to a decrease of electron density in the N−H bond. As a result, the N−H-bond is weakened and the frequency of the respective vibration is shifted to lower values. Therefore, δ(N−H)-bending vibration band is a good reporter of the dehydration of the polymer network at the volume phase transition [51]. All ${\tilde{\nu }}_{\max}\left(t\right)$-swelling curves show one smooth phase transition which is an evidence for a statistical distribution of monomers in the polymer network. Furthermore, the continuous curves again indicate a cooperativity of the phase transition, see Figure 6. The description of the ${\tilde{\nu }}_{\max}\left(t\right)$-swelling curves is given by the Hill-like model applied to this case:

(14) $${\tilde{\nu }}_{\max}={\tilde{\nu }}_{\max;0}+\sum_{i}x_{i}\left(a_{i}\left(t-t_{a}\right)+b_{i}\frac{t_{rel}^{\nu_{i}}}{t_{rel}^{\nu_{i}}+{\left(\frac{VPTT_{fit}-t_{a}}{t_{e}-t_{a}}\right)}^{\nu_{i}}}\right)$$

where ${\tilde{\nu }}_{\max;0}$ is the value of the maximum of the δ(N−H)-bending vibration band at temperature $t_{a}$, $x_{i}$ is the molar fraction, $a_{i}$ is the slope of the baseline and $b_{i}$ is the amplitude parameter of the Hill transition. The index *i* stands for a polymer component which in this work can be NNPAM, NIPAM or NIPMAM. For the fit $\nu_{i}$ values were taken from the previous work [35] for the respective components, see Table 2.

The parameters $a_{i}$ were determined from the fit of the ${\tilde{\nu }}_{\max}\left(t\right)$-swelling curves of the homopolymers. For the copolymer systems as well as for the homopolymer systems the fitting parameters $b_{i}$, and $VPTT_{fit}$ are shown in Table 3. The fits were successful in all cases which is also confirmed by the small ${\left(chi\right)}^{2}$ values. It should be emphasized that the Hill parameter $\nu_{i}$ from the previous work [35] leads to good fitting results. The parameter $b_{i}$ changes depending on the composition instead of being constant as in the $R_{H}\left(t\right)$-swelling curves fits.

A reason for this is that the description of the $R_{H}\left(t\right)$-swelling curves takes into account the interaction between polymer and solvent of the whole polymer network, and the description of the ${\tilde{\nu }}_{\max}\left(t\right)$-swelling curves takes into account only the interaction between the solvent and the (N−H)-bond. The $VPTT_{fit}$ resulting from the analysis corresponds to the $VPTT_{IP}$ determined from the inflection point of the experimental curve, see Table 3 and Figure 3. The VPTT of the copolymer systems show a linear trend between the homopolymer systems, which further supports a statistical nature and cooperativity of the volume phase transition. The fact that VPTT are linearly correlated with nominal composition is consistent with the results of other works [18,27,28,30,52,53].

This linear relationship reflects the change in hydrophilicity with the comonomer content. If the hydrophilicity of the polymer network increases, the VPTT also increases.

## 4. Conclusions

Copolymer microgels with different comonomer compositions were synthesized by precipitation polymerization and characterized by PCS and FTIR spectroscopy. The comonomer content in the microgels was successfully determined by ^1^H-NMR spectroscopy. The results show that the determined comonomer content is in agreement within the error margin of 5 mol% with the nominal comonomer feed used in synthesis. We have shown that the $R_{H}\left(t\right)$-swelling curves obtained by PCS can be quantitatively described using the Flory–Rehner theory for copolymers introduced by Godbole et al. [40] modiefied by the Hill-like model for the interaction parameter χ. For the analysis, the fixed parameters $\chi_{0,i}$, $a_{i}$, $b_{i}$ and $\nu_{i}$ were used which are universally applicable for the respective monomers given in our previous work [35]. It is remarkable that only the three fitting parameters $\phi_{0}$, $N_{Seg}$, and $VPTT_{fit}$ are needed to describe a rather complex copolymer microgel. The calculated swelling curves yield physically reasonable and meaningful results. Using this description, the homopolymer systems as well as the copolymer systems could be described quantitatively. In addition, ${\tilde{\nu }}_{\max}\left(t\right)$-swelling curves from FTIR spectroscopy also were successfully fitted using the Hill-like model. The obvious cooperativity of the volume phase transition in homopolymer microgels and statistical copolymer microgels were successfully taken into account by using the Hill-like model. In the future, the universal monomer specific parameters $\chi_{0,i}$, $a_{i}$, $b_{i}$ and the concentration dependent parameter $\nu_{i}$ can be used as reference parameters for fitting different homopolymer and copolymer systems. The generalized Flory–Rehner theory [40] using the Hill-like model [35,36,38] needs just three fitting parameters $\phi_{0}$, $N_{Seg}$, and $VPTT_{fit}$ to describe the swelling curves of various copolymer microgels for which the swelling behavior of the respective homopolymer is already known.

## 5. Materials and Methods

### 5.1. Materials

*N-n*-propylacrylamide (NNPAM) was synthesized via a Schotten–Baumann reaction published by Hirano et al. [54]. For this reaction, acryloylchloride (Sigma-Aldrich Chemie GmbH, Munich, Germany; purity 98%), *n*-propylamine (Fluka, Sigma-Aldrich Chemie GmbH, Munich, Germany; purity 99%), triethylamine (Grüssing GmbH Analytika, Filsum, Germany; purity 99%), and methylenechloride (p.a.) were used as received. The obtained monomer NNPAM was washed with $NaHCO_{3}$ (10 wt%) and dried over $MgSO_{4}$. After filtration, the solvent was evaporated and the product was distilled in vacuum (115 °C, 10 mbar). *N*-isopropylacrylamide (NIPAM; Sigma-Aldrich Chemie GmbH, Munich, Germany; purity 97%) and *N*-isopropylmethacrylamide (NIPMAM; Sigma-Aldrich Chemie GmbH, Munich, Germany; purity 97%) were purified by recrystallization from hexane. The cross-linker *N,N’*-methylenebisacrylamide (BIS; Sigma-Aldrich Chemie GmbH, Munich, Germany; purity 99%) and the initiator ammonium persulfate (APS; Sigma-Aldrich Chemie GmbH, Munich, Germany; purity ≥98%) were used without further purification. For all experiments, purified water from an Arium pro VF system (Sartorius AG, Göttingen, Germany) was used.

### 5.2. Synthesis of Homo- and Copolymer Microgels

Homopolymer microgels poly(NNPAM), poly(NIPAM), and poly(NIPMAM) and copolymer microgels poly(NNPAM-co-NIPMAM), poly(NNPAM-co-NIPAM) and poly(NIPAM-co-NIPMAM) were synthesized via conventional precipitation polymerization without surfactant, (Table 4). All syntheses were performed in 100 mL three-neck flasks equipped with a reflux condenser, mechanical stirrer (400 rpm), and a nitrogen inlet. The monomers (3.85 mmol) and the cross-linker *N,N’*-methylenebisacrylamide (BIS) (3.85 × 10^−1^ mmol; 10.0 mol% respective to the total monomer amount) were dissolved in 50 mL purified water and heated to 70 °C under continuous stirring and purged with nitrogen. After 1 h the polymerization was initiated by the addition of 1 mL of the 38.5 mM solution of APS (1.0 mol% respective to the total monomer amount) and left to proceed for 4 h at 70 °C. Subsequently, the solution was cooled to room temperature and stirred overnight. For purification, all samples were treated by five cycles of centrifugation, decantation, and redispersion in purified water using a 70 Ti rotor in an Optima L-90K centrifuge (Beckman Coulter GmbH, Krefeld, Germany) at 20,000 rpm and 25 °C.

### 5.3. Photon Correlation Spectroscopy

Temperature-dependent measurements were performed with a PCS setup, consisting of a cw-laser (532 nm, MGI-FN-532-100 mW), a multiple-τ digital correlator (ALV/LSE-5004, ALV-GmbH, Langen, Germany), a single photon detector (ALV/SO-SIPD Single Photon Detector, ALV-GmbH, Langen, Germany) and a laser goniometer (ALV/SP-86, ALV-GmbH, Langen, Germany). The sample was tempered in a decalin index-matching-bath and equilibrated at the desired temperature for 25 min. At each temperature, five measurements of 200 s at a scattering-angle of $\theta =45^{\circ }$ in pseudo-cross-correlation-mode were performed. With the obtained mean relaxation rates $\bar{\Gamma }$ of the $g^{1}\left(t\right)$ functions the hydrodynamic radii $R_{H}$ were calculated by the Stokes-Einstein relation (Equation (15)):

(15) $$R_{H}=\frac{k_{B}T}{6\pi \eta \frac{\bar{\Gamma }}{q^{2}}}$$

where, $k_{B}$ is the Boltzmann constant, η the solvent viscosity (water), *T* the temperature and $q=\frac{4\pi n}{\lambda }\sin\frac{\theta }{2}$ the magnitude of the scattering vector with the refractive index of the solvent *n*.

### 5.4. Fourier Transformation Infrared Spectroscopy

For the temperature-dependent FTIR measurements a Tensor 27 FTIR spectrometer (Bruker, Ettlingen, Germany) in transmission mode with a tailor-made BaF_2_ cuvette (Korth Kristalle, Kiel, Germany) was used. At each temperature the absorbance spectra were calculated from the microgel spectrum and as reference the spectrum of H_2_O whereby the water absorbance was corrected for algorithmically as described before [51]. Afterwards, the maximal frequencies of the temperature-dependent NH-bands were extracted and plotted versus the temperature. To quantify the phase transition the data were fitted with a Hill-like function, Equation (14).

### 5.5. Nuclear Magnetic Resonance Spectroscopy

For sample preparation, 5 mg of the freeze-dried microgel was dispersed in D_2_O and was transferred into a NMR tube (Boroeco-5-7, Deutero GmbH, Kastellaun, Germany). ^1^H-NMR spectra were measured on an Avance III 500 (500 MHz) (Bruker Corporation) at 298 K. As reference the proton signal of D_2_O ( δ = 4.79 ppm) was used. Spectra were corrected for phase and baseline (Whittaker Smoother) prior to integration of signals. 

## Figures and Tables

**Table 1:** Parameters resulting from the fit of the presented swelling curves and the corresponding ${\left(chi\right)}^{2}$-values of the fits and the volume phase transition temperature ($VPTT_{IP}$) determined from the inflection point of the $R_{H}\left(t\right)$–swelling curve.

System	Nominal Comonomer Content /mol%	Measured Comonomer Content /mol%	$\phi_{0}$	$N_{\mathrm{Seg}}$	${\mathrm{VPTT}}_{\mathrm{fit}}$ /°C	${\mathrm{VPTT}}_{\mathrm{IP}}$ /°C	${\left(\mathrm{chi}\right)}^{2}$ /nm
poly(NNPAM)	–	–	0.77	46	23.2	22.2	0.9
poly(NIPAM)	–	–	0.84	51	35.1	33.4	0.3
poly(NIPMAM)	–	–	0.71	43	43.3	41.8	1.0
poly(NNPAM-co-NIPAM)	30.0	30.1	0.78	60	28.1	26.7	0.3
	50.0	54.3	0.74	44	29.5	28.7	0.5
	70.0	74.9	0.74	45	31.7	30.8	0.4
poly(NIPAM-co-NIPMAM)	30.0	34.0	0.89	63	37.9	35.9	1.0
	50.0	50.7	0.78	46	39.2	37.6	0.8
	70.0	73.1	0.82	53	40.6	40.7	2.7
poly(NIPMAM-co-NNPAM)	30.0	26.3	0.75	29	36.3	36.2	1.9
	50.0	51.2	0.83	45	32.6	31.3	0.4
	70.0	74.6	0.77	49	28.4	27.4	1.1

**Table 2:** Parameters of the homopolymers determined from our previous work [35]. The parameters $\chi_{0}$, *a* and *b* are independent of the cross-linker *N,N’*-methylenebisacrylamide (BIS) and initiator ammonium persulfate (APS) concentration but ν is dependent on the BIS concentration. For all syntheses, a BIS concentration of 10 mol% was used therefore ν at 10 mol% BIS from the previous work is listed here. ν nicely follows the hydrophilicity difference of the three systems with poly(NIPMAM) being the most hydrophilic.

System	$\chi_{0}$	*a*	*b*	ν
		/K ^−1^		
poly(NNPAM)	0.239	0.012	0.576	8.10
poly(NIPAM)	0.020	0.020	0.290	15.5
poly(NIPMAM)	−0.074	0.016	0.387	20.2

**Table 3:** Parameters resulting from the fit of ${\tilde{\nu }}_{\max}\left(t\right)$-swelling curves and the corresponding ${\left(chi\right)}^{2}$-values of the fits and the volume phase transition temperatures ($VPTT_{IP}$) determined from the inflection point of the ${\tilde{\nu }}_{\max}\left(t\right)$-swelling curves. The parameters $a_{i}$ were kept constant for the fits ($a_{NNPAM}$ = −0.186 ${\mathrm{cm}}^{-1}K^{-1}$; $a_{NIPAM}$ = −0.211 ${\mathrm{cm}}^{-1}K^{-1}$; $a_{NIPMAM}$ = −0.155 ${\mathrm{cm}}^{-1}K^{-1}$).

System	Measured Comonomer Content/mol%	$b_{\mathrm{NNPAM}}$/${\mathrm{cm}}^{-1}$	$b_{\mathrm{NIPAM}}$/${\mathrm{cm}}^{-1}$	$b_{\mathrm{NIPMAM}}$/${\mathrm{cm}}^{-1}$	${\mathrm{VPTT}}_{\mathrm{fit}}$/°C	${\mathrm{VPTT}}_{\mathrm{IP}}$/°C	${\left(\mathrm{chi}\right)}^{2}\times 10^{4}$/${\mathrm{cm}}^{-1}$
poly(NNPAM)	–	−3.907	–	–	22.7	22.2	3.8
poly(NIPAM)	–	–	−3.140	–	34.8	34.9	1.2
poly(NIPMAM)	–	–	–	−3.999	42.0	41.0	1.8
poly(NNPAM-co-NIPAM) ^1^	30.1	−3.891	−3.146	–	27.2	26.8	1.2
	54.3	−2.165	−3.143	–	29.4	29.2	0.6
	74.9	−3.958	−3.181	–	32.6	31.8	3.4
poly(NIPAM-co-NIPMAM)	34.0	–	−3.213	−2.983	36.4	37.4	4.0
	50.7	–	−2.489	−3.152	38.9	38.3	4.5
	73.1	–	−2.797	−3.030	40.2	40.1	4.2
poly(NIPMAM-co-NNPAM)	26.3	−4.947	–	−2.657	36.5	36.2	0.3
	51.2	−0.878	–	−3.685	33.0	31.6	8.3
	74.6	−2.053	–	−4.169	28.9	27.5	7.1

^1^ For poly(NNPAM-co-NIPAM) with a comonomer content of 74.9 mol% aNIPAM = −1.68 cm^−1^K^−1^ was used because the fit was poor with $a_{NNPAM}$ = −0.211 cm^−1^K^−1^.

**Table 4:** List of the molar quantities of the monomers used for the respective homo- and copolymer microgels synthesized. The nominal comonomer content is related to the total amount of monomers used.

System	Nominal Comonomer Content /mol%	[NNPAM] /mmol	[NIPAM] /mmol	[NIPMAM] /mmol
poly(NNPAM)	–	3.850	–	–
poly(NIPAM)	–	–	3.850	–
poly(NIPMAM)	–	–	–	3.850
poly(NNPAM-co-NIPAM)	30	2.695	1.155	–
	50	1.925	1.925	–
	70	1.155	2.695	–
poly(NIPAM-co-NIPMAM)	30	–	2.695	1.155
	50	–	1.925	1.925
	70	–	1.155	2.695
poly(NIPMAM-co-NNPAM)	30	1.155	–	2.695
	50	1.925	–	1.925
	70	2.695	–	1.155

## Data Availability

Data can be obtained from the authors upon request.

## Supplementary Materials

The following are available online at https://www.mdpi.com/article/10.3390/polym14101999/s1, Table S1: List of measured comonomer contents of poly(NNPAM-co-NIPAM), poly(NIPAM-co-NIPMAM), and poly(NIPMAM-co-NNPAM), Figure S1: ^1^H-NMR spectrum of microgel poly(NNPAM), Figure S2: ^1^H-NMR spectrum of microgel poly(NIPAM), Figure S3: ^1^H-NMR spectrum of microgel poly(NIPMAM), Figure S4: ^1^H-NMR spectrum of microgel poly(NNPAM), poly(NIPAM) and poly(NNPAM-co-NIPAM) with different comonomer contents, Figure S5: ^1^H-NMR spectrum of microgel poly(NIPAM), poly(NIPMAM) and poly(NIPAM-co-NIPMAM) with different comonomer contents, Figure S6: ^1^H-NMR spectrum of microgel poly(NIPMAM), poly(NNPAM) and poly(NIPMAM-co-NNPAM) with different comonomer contents.
