# Supplementary material for: Modified Flory–Rehner Theory Describes Thermotropic Swelling Transition of Smart Copolymer Microgels
Source: Polymers (Basel). 2022 May 13;14(10):1999. doi: 10.3390/polym14101999 (PMC9143634; doi:10.3390/polym14101999)
Supplement: Supplementary file 1 [file polymers-14-01999-s001.zip › polymers-1713442-Supplementary.pdf]

polymers

MDPI

# Supplementary Materials: Modified Flory-Rehner Theory Describes Thermotropic Swelling Transition of Smart Copolymer Microgels

**Simon Friesen, Sergej Kakorin and Thomas Hellweg ***

## 1. $^1$H-NMR analysis

Quantification of the comonomer content was carried out using $^1$H-NMR spectra. A three-fold quantification was performed. The results are listed in Table S1. For the calculation of the comonomer content in p(NNPAM-co-NIPAM) and p(NIPMAM-co-NNPAM) microgels, the signal of the isopropyl H atom ($\delta$ = 3.93 ppm NHC<u>H</u>($CH_3$)$_2$, 1H) and the signal of the H atoms of the secondary carbon ($\delta$ = 3.14 ppm NHC<u>H$_2$</u>$CH_2CH_3$, 2H) were used. From the ratios of the intensity integrals of the signals, the comonomer content was calculated. Since all signals overlap in p(NIPAM-co-NIPMAM) microgels, the signals from the H atoms of the methyl groups ($\delta \approx$ 1.15 ppm) of the homopolymer microgels p(NIPAM) and p(NIPMAM) and copolymer microgels p(NIPAM-co-NIPMAM) were normalized to the signal of the isopropyl H atom ($\delta \approx$ 3.93 ppm). Linear combination of the normalized values of the homopolymers were used to calculate the comonomer content in the copolymer microgels.

**Table S1.** Nominal comonomer content and measured comonomer content of p(NNPAM-co-NIPAM), p(NIPAM-co-NIPMAM) and p(NIPMAM-co-NNPAM). The copolymerization ratio was calculated from the $^1$H-NMR spectra.

| nominal comonomer content / mol% | p(NNPAM-co-NIPAM) measured comonomer content / mol% | p(NIPAM-co-NIPMAM) measured comonomer content / mol% | p(NIPMAM-co-NNPAM) measured comonomer content / mol% |
|---|---|---|---|
| 30.0 | 30.2 | 36.9 | 26.2 |
| | 30.0 | 33.6 | 26.2 |
| | 30.0 | 31.4 | 26.5 |
| 50.0 | 55.1 | 51.1 | 44.9 |
| | 54.3 | 50.4 | 45.7 |
| | 53.5 | 50.7 | 46.5 |
| 70.0 | 74.9 | 72.3 | 69.8 |
| | 74.9 | 73.3 | 70.0 |
| | 74.9 | 73.7 | 70.0 |

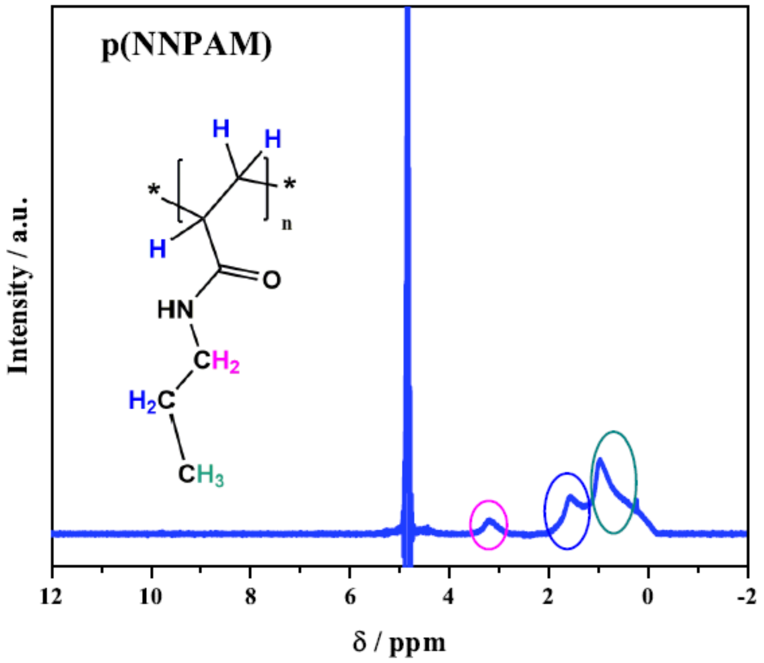


**Figure S1.** $^{1}$H-NMR spectrum of microgel p(NNPAM) and the chemical structural formula with the H atoms highlighted in color. *δ* = 0.91 ppm ($NHCH_2CH_2C\underline{H_3}$, 3H) (green); *δ* ≈ 1.50 ppm ($NHCH_2C\underline{H_2}CH_3$, 2H) (blue); *δ* ≈ 1.50 ppm ($C\underline{H}CH_2$, 1H) (blue); *δ* ≈ 1.50 ppm ($CHC\underline{H_2}$, 2H) (blue); *δ* = 3.14 ppm ($NHC\underline{H_2}CH_2CH_3$, 2H) (pink).

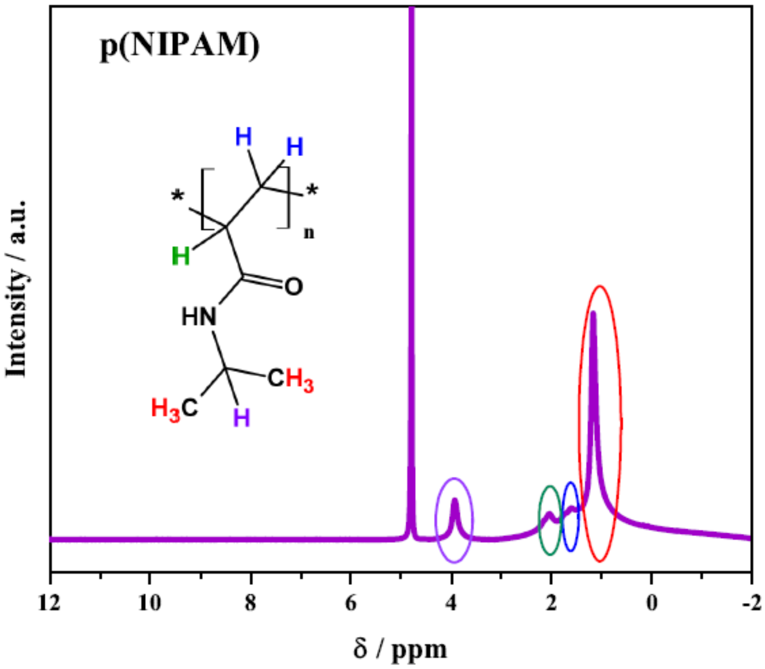


**Figure S2.** $^{1}$H-NMR spectrum of microgel p(NIPAM) and the chemical structural formula with the H atoms highlighted in color. *δ* = 1.17 ppm ($NHCH(C\underline{H_3})_2$, 6H) (red); *δ* = 1.61 ppm ($CHC\underline{H_2}$, 2H) (blue); *δ* = 2.05 ppm ($C\underline{H}CH_2$, 1H) (green); *δ* = 3.93 ppm ($NHC\underline{H}(CH_3)_2$, 1H) (purple).

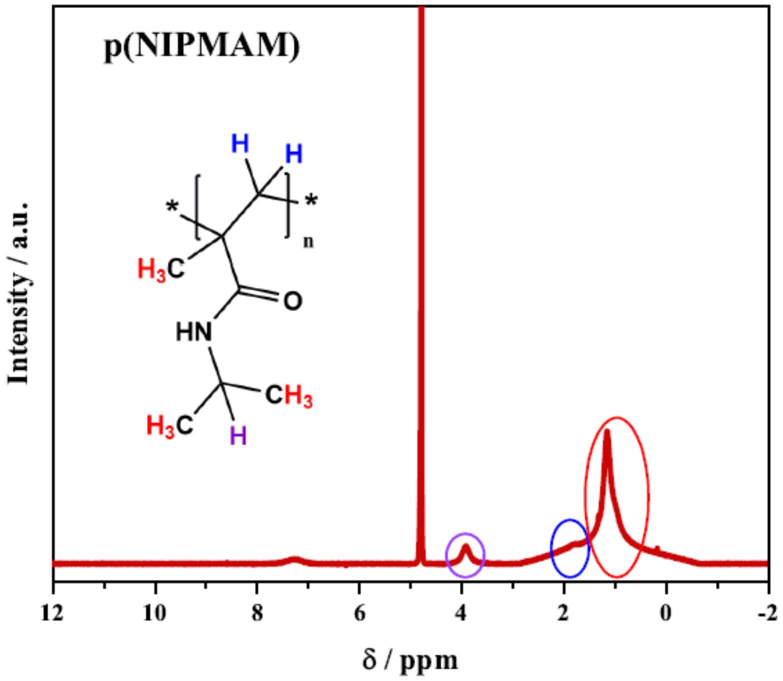


**Figure S3.** $^{1}$H-NMR spectrum of microgel p(NIPMAM) and the chemical structural formula with the H atoms highlighted in color. $\delta \approx$ 1.15 ppm (NHCH(C<u>H</u>3)2, 6H) (red); $\delta \approx$ 1.15 ppm (CC<u>H</u>3CH2, 3H) (red); $\delta \approx$ 1.78 ppm (CCH3C<u>H</u>2, 2H) (blue); $\delta$ = 3.92 ppm (NHC<u>H</u>(CH3)2, 1H) (purple).

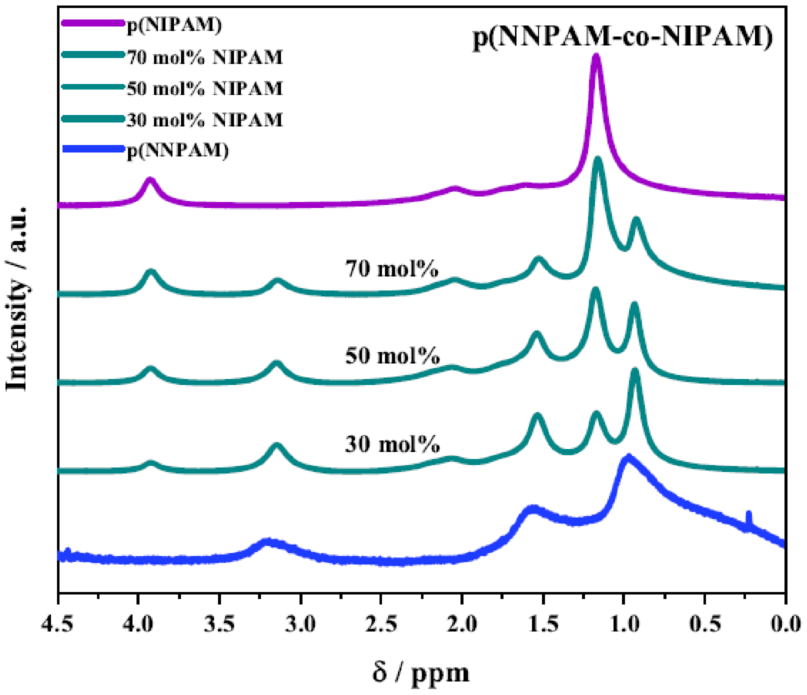


**Figure S4.** $^{1}$H-NMR spectrum of microgel p(NNPAM), p(NIPAM) and p(NNPAM-co-NIPAM) withdifferent comonomer contents.

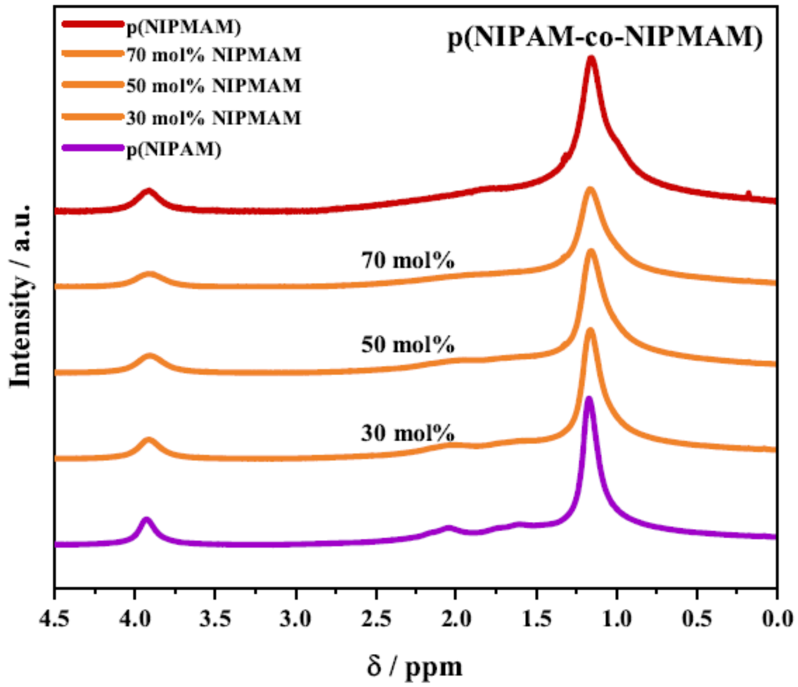


**Figure S5.** $^{1}$H-NMR spectrum of microgel p(NIPAM), p(NIPMAM) and p(NIPAM-co-NIPMAM)with different comonomer contents.

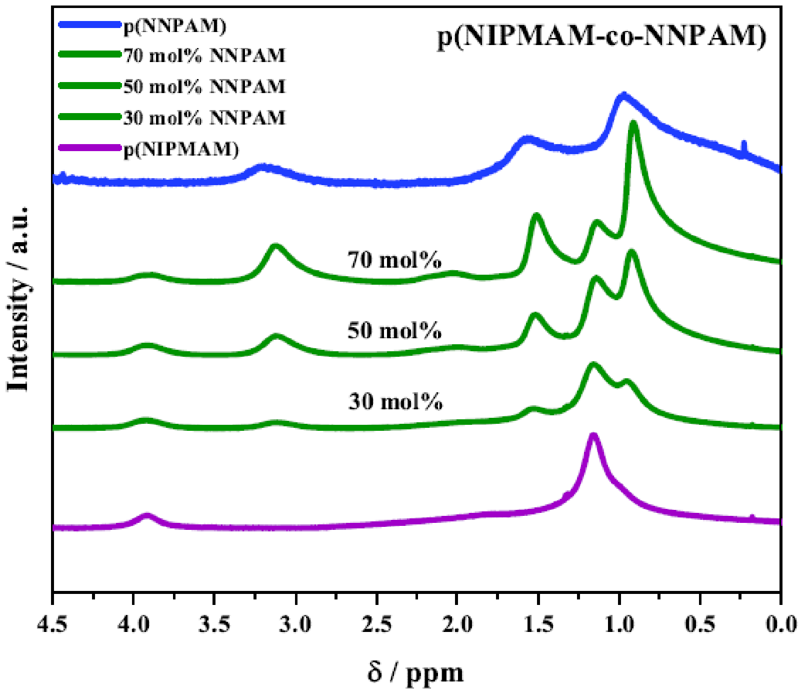


**Figure S6.** $^{1}$H-NMR spectrum of microgel p(NIPMAM), p(NNPAM) and p(NIPMAM-co-NNPAM)with different comonomer contents.
